# *Enterocytozoon bieneusi* in Minks (*Neovison vison*) in Northern China: A Public Health Concern

**DOI:** 10.3389/fmicb.2018.01221

**Published:** 2018-06-12

**Authors:** Xiao-Xuan Zhang, Ruo-Lan Jiang, Jian-Gang Ma, Chao Xu, Quan Zhao, Guangyu Hou, Guo-Hua Liu

**Affiliations:** ^1^Hunan Provincial Key Laboratory of Protein Engineering in Animal Vaccines, College of Veterinary Medicine, Hunan Agricultural University, Changsha, China; ^2^College of Animal Science and Veterinary Medicine, Heilongjiang Bayi Agricultural University, Daqing, China; ^3^School of Basic Medical Sciences, Xiangya School of Medicine, Central South University, Changsha, China; ^4^College of Veterinary Medicine, Northwest A&F University, Yangling, China; ^5^Key Laboratory of Agricultural Ministry, State Key Laboratory of Special Economic Animal Molecular Biology, Institute of Special Animal and Plant Sciences of Chinese Academy of Agricultural Sciences, Changchun, China; ^6^College of Animal Science and Technology, Changchun Sci-Tech University, Changchun, China; ^7^College of Basic Medicine, Mudanjiang Medical College, Mudanjiang, China

**Keywords:** *Enterocytozoon bieneusi*, prevalence, minks, genotyping, Northern China

## Abstract

*Enterocytozoon bieneusi* is the most important causative agent of microsporidiosis and can infect almost all vertebrate and invertebrate hosts, including minks (*Neovison vison*). In the present study, a total of 298 feces samples (including 79 from Heilongjiang province, 31 from Hebei province, 67 from Jilin province, 90 from Liaoning province, and 31 from Shandong province, Northern China) were examined by nested PCR amplification of the internal transcribed spacer (ITS) region of the rRNA gene. The overall prevalence of *E. bieneusi* in minks was 10.1%, with 10.5% in Jilin province, 32.3% in Hebei province, 8.9% in Liaoning province, 0% in Shandong province, and 6.3% in Heilongjiang province. Furthermore, multiple logistic regression analysis revealed that region was only risk factors associated with *E. bieneusi* infection in the investigated minks. Five *E. bieneusi* ITS genotypes (three known genotypes, namely D, Peru11, and EbpC; two novel genotypes, namely, NCM-1 and NCM-2) were found in the current study. Importantly, genotypes D, Peru11 and EbpC, previously identified in humans, were also found in minks, which suggested that minks are the potential sources of human microsporidiosis. To the best of our knowledge, this is the first report of *E. bieneusi* infection in minks worldwide. The results of the present survey have implications for the controlling *E. bieneusi* infection in minks, other animals and humans.

## Introduction

*Enterocytozoon bieneusi* is the most frequently detected Microsporidia species, which composed of over 1300 named species, classified into 160 genera (da Cunha et al., 2016; Li et al., 2016a). It is ubiquitous in the environment and is responsible for over 90% of intestinal microsporidiosis in humans (Desportes et al., 1985). Transmission of *E. bieneusi* was mainly through fecal-oral route, such as ingestion of food and/or water contaminated by spores of *E. bieneusi* (Prasertbun et al., 2017). AIDS infection with this pathogen may cause life-threatening chronic diarrhea (Canning and Hollister, 1990). Although immunocompetent individual infection with *E. bieneusi* are usually asymptomatic, they can shed spores into the environment (Zhao et al., 2015).

To date, more than 240 *E. bieneusi* genotypes were defined based on the internal transcribed spacer (ITS) region of the rRNA gene (da Cunha et al., 2016), which were classified into nine groups by phylogenetic analyses. Among these groups, group 1 representing zoonotic potential phylogenetic group was responsible for the majority of human infections while the remainings (groups 2 to 9) were host-adapted phylogenetic groups, which more frequently recorded in specific hosts or water (Hu et al., 2014; Ma et al., 2015). Interestingly, several genotypes, such as I, J, and BEB4 were divided into phylogenetic groups 2, but they have also been found in humans (Jiang et al., 2015), indicating that these genotypes have zoonotic potential. Therefore, there are raised some questions in the genotypes identification of *E. bieneusi*.

*Enterocytozoon bieneusi* was firstly identified in pig feces (Deplazes et al., 1996). Many animals have been now recognized as its hosts (Du et al., 2015; Qi et al., 2015; Fiuza et al., 2016; Li et al., 2016b; Xu et al., 2016; Zhang et al., 2016a,b; da Cunha et al., 2017; Yue et al., 2017). Although *E. bieneusi* from other animals can be a potential source for human infections (Xu et al., 2016; Zhang et al., 2016a; Yue et al., 2017), no information is available about prevalence and genotypes of *E. bieneusi* in minks. Therefore, in the present study, a total of 298 feces samples from five provinces of Northern China were examined to estimate the *E. bieneusi* prevalence and to identify their genotypes. Our results should provide a foundation for the improved control of *E. bieneusi* infection in humans and animals in these regions and elsewhere in China.

## Materials and Methods

### Ethics Approval and Consent to Participate

This study was approved by the Animal Ethics Committee of Hunan Agricultural University. Minks used for the study were handled in accordance with good animal practices required by the Animal Ethics Procedures and Guidelines of the People’s Republic of China.

### Specimen Collection

A total of 298 fecal samples were randomly collected from nine farmed minks (randomly collected) in Jilin province (41°∼46° N, 122°∼131° E), Liaoning province (38°∼43° N, 118°∼125° E), Heilongjiang province (43°26′∼53°33′ N, 121°11′∼135°05′ E), Hebei province (36°05′∼42°40′ N, 113°27′∼119°50′ E), and Shandong province (34°22.9′∼38°24.01′ N, 114°47.5′∼122°42.3′ E), Northern China in 2016 (**Table 1**). The numbers of minks reared on each farm ranged from 300 to 1800, approximately. From each farm, approximately 3% of animals was sampled. Before sampling, animals were subjected to clinical examination to determine their health status. Each fecal sample (approximately 50* g*) was collected using sterile gloves immediately after the animal had defecated, and then was placed into ice boxes and quickly transported to the laboratory. Information about each mink, such as season (missing the spring), gender, geographic origin, age, and farm ID were collected.

**Table 1:** Prevalence of *Enterocytozoon bieneusi* in minks in Heilongjiang, Hebei, Jilin, Liaoning, and Shandong provinces, Northern China.

Factor	Category	No. of tested	No. of positive	Prevalence (%) (95% CI)	*P*-value	OR (95% CI)
Region	Heilongjiang province	79	5	6.3% (0.8–11.8)	0.01	Reference
	Hebei province	31	10	32.3% (14.8–49.7)		7.1 (2.2–22.9)
	Jilin province	67	7	10.5% (2.9–18.0)		1.7 (0.5–5.7)
	Liaoning province	90	8	8.9% (2.9–14.9)		1.4 (0.5–4.6)
	Shandong province	31	0	0 (–)		–
Gender	Female	145	13	9.0% (4.3–13.7)	0.54	Reference
	Male	153	17	11.1% (6.1–16.1)		1.3 (0.6–2.7)
Age	≤3 months	148	13	8.8% (4.2–13.4)	0.47	Reference
	>3 months	150	17	11.3% (6.2–16.5)		1.3 (0.6–2.8)
Season	Winter	57	4	7.0% (0.2–13.9)	0.31	Reference
	Autumn	93	13	14.0% (6.8–21.2)		2.2 (0.7–7.0)
	Summer	148	13	8.8% (4.2–13.4)		1.3 (0.4–4.1)
Total		298	30	10.1% (6.6–13.5)		


### DNA Extraction, PCR Amplification, and Sequencing

Genomic DNA was extracted using the E.Z.N.A.^®^ Stool DNA Kit (Omega Bio-tek Inc., Norcross, GA, United States) (Xu et al., 2016). Then DNA samples were stored at -20°C until tested. The molecular identity and genotype of *E. bieneusi* of each specimen was detected by PCR-based sequencing of ITS locus using previous established methods (Xu et al., 2016; Zhang et al., 2016a). PCR products were send to Sangon Biotech Company (Shanghai, China) for sequencing from both directions.

### Phylogenetic Analyses

The ITS locus sequences of the representative samples (representing different genotypes) were used for phylogenetic analyses. The obtained ITS sequences were aligned with the corresponding reference sequences in GenBank using ClustalX1.81 (Thompson et al., 1997). The neighbor-joining (NJ) were performed using Mega 5.0 (Tamura et al., 2011). The Kimura 2-parameter model were selected as the most suitable model. NJ tree were calculated based on 1,000 bootstrap replicates.

### Statistical Analysis

The variation in prevalence of *E*. *bieneusi*-infected minks (*y*) of age (*x*1), gender (*x*2), different geographical location (*x*3), and season (*x*4) were analyzed by χ^2^ test using SAS version 9.1 (SAS Institute, Cary, NC, United States) (Meng et al., 2017; Zhang et al., 2017). Each of these variables was included in the binary logit model as an independent variable by multivariable regression analysis. When *P* < 0.05, the results were considered statistically significant. The adjusted odds ratio (OR) and 95% confidence interval (CI) for each variable were calculated with binary logistic regression and all risk factors entered simultaneously.

## Results

### Prevalence of *E. bieneusi*

The study showed that 30 (10.1%, 95% CI 6.6–13.5) of the 298 tested fecal samples were positive for *E*. *bieneusi* (**Table 1**). The prevalence of *E*. *bieneusi* in different region groups ranged from 0.0 to 32.3% (**Table 1**). Of the nine farms, six farms were detected *E. bieneusi*-positive, with the highest prevalence in farm 3 (32.3%) located in Hebei province (**Table 2**). Furthermore, prevalence of *E. bieneusi* in minks of less than 3 months old and minks of more than 3 months old was 8.8 and 11.3%, respectively (*P* = 0.47). The prevalence of *E. bieneusi* in female minks (9.0%) was lower than males (11.1%) (*P* = 0.54). Prevalence of *E. bieneusi* was 8.8% in summer, 14.0% in autumn and 7.0% in winter (*P* = 0.31). Moreover, a significant correlation between the investigated region and *E. bieneusi* infection (*P* = 0.01) was observed by logistic regression analysis.

**Table 2:** Distribution of *Enterocytozoon bieneusi* genotypes in different farms.

Region	Farm ID	Sample size	Prevalence (%)	Genotypes (no.)
Jilin province	1	9	0	–
	2	58	12.1%	NCM-1 (4); D (3)
Hebei province	3	31	32.6%	Peru11 (1); D (5); EbpC (4)
Liaoning province	4	30	10.0%	Peru11 (3)
	5	30	16.7%	D (2); EbpC (3)
	6	30	0	–
Shandong province	7	31	0	–
Heilongjiang province	8	50	8.0%	NCM-2 (1); NCM-1 (1); D (2)
	9	29	3.5%	Peru 11 (1)
Total		298	10.1%	D (12); Peru 11 (5); EbpC (7); NCM-1 (5); NCM-2 (1)


### Genetic Characterizations and Genotype Distribution of *E. bieneusi* in Minks

DNA sequence analysis of the ITS locus suggested five *E. bieneusi* ITS genotypes (three known genotypes and two novel genotypes) were identified in this study, namely, D, Peru11, EbpC, NCM-1, and NCM-2 (**Table 2**). Of these genotypes, D was the predominance genotype which is present in four farms (*n* = 12), was responsible for 40.0% of all infection; genotype EbpC (*n* = 7, 23.3%) and NCM-1 (*n* = 5, 16.7%) were all found in two farms; genotype Peru11 (*n* = 5, 16.7%) was found in three farms; NCM-2 (*n* = 1, 3.3%) was only identified in farm 8 located in Heilongjiang province. Moreover, a total of seven polymorphic sites were observed among the five genotypes (**Table 3**). Phylogenetic analysis of ITS sequences indicated that the three known genotypes sequences (accession nos: MF440664, MF440667, and MF440668) were identical to that of genotypes D (accession no. KT922238), Peru11 (accession no. JX994269), and EbpC (accession no. KR815517) sequences, respectively.

**Table 3:** Variations in the ITS nucleotide sequences among genotypes of the *Enterocytozoon bieneusi* in minks in Northern China.

Genotypes (no.)	Nucleotide at position							GenBank accession nos.
	106	158	178	182	195	206	254	
D (12)	A	C	C	T	A	T	A	MF440664
NCM-1 (5)	G	T	T	G	G	C	A	MF440665
NCM-2 (1)	A	T	T	G	G	C	G	MF440666
Peru11 (5)	A	C	C	T	A	T	A	MF440667
EbpC (7)	A	T	T	G	G	C	A	MF440668


### Phylogenetic Relationship of *E. bieneusi*

**FIGURE 1:**
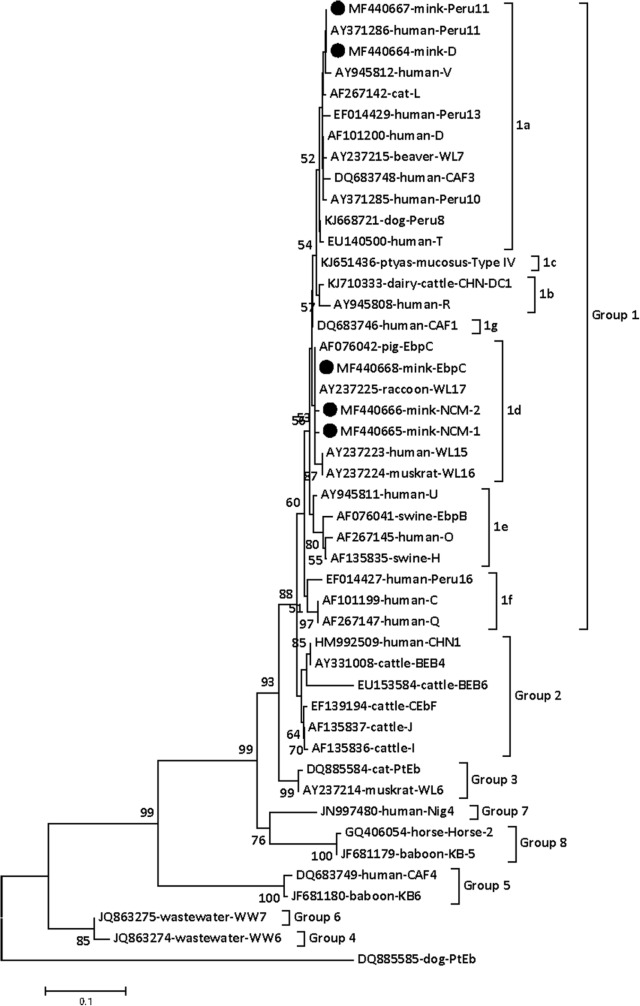
Phylogenetic analyses of *Enterocytozoon bieneusi* using neighbor-joining (NJ). *E. bieneusi* isolates identified in the present study are pointed out by solid circles.

To ascertain the identity of the *E. bieneusi* isolates, phylogenetic relationship of *E. bieneusi* isolates were reconstructed base on the ITS sequences. In this tree, groups 1, 2, 3, 5 were in different clade, respectively; however, groups 7, 8 and groups 4, 6 were in two different clade, respectively (**Figure 1**). Group 1 and group 2 were more closely related than to other groups. These results indicated that all the *E. bieneusi* isolates in present study were classified to group 1 (**Figure 1**). Group 1 can be further divided into six subgroup (subgroups 1a–f). D and Peru11 were in supgroup 1a (**Figure 1**); however, EbpC, NCM-1, and NCM-2 were in subgroup 1d (**Figure 1**).

## Discussion

*Enterocytozoon bieneusi* is an important enteric pathogen. Humans acquired *E. bieneusi* infection may present with the symptoms of diarrhea and enteric diseases (Zhao et al., 2015). Various studies have been reported from *E. bieneusi* prevalence in different animals around the world (Santín and Fayer, 2011; Ma et al., 2015; Santin and Fayer, 2015; Prasertbun et al., 2017), but information about distribution of genotypes of *E. bieneusi* in captive animals in China is scarce, especially in minks.

In the present study, the overall prevalence of *E. bieneusi* in minks was 10.1% (30/298), which was higher than that in domestic rabbits (0.94%, 4/426) (Zhang et al., 2016b), raccoon dogs (4.1%, 2/49) (Zhao et al., 2015), pet chinchillas (3.6%, 5/140) (Qi et al., 2015), captive snakes (4.6%, 11/240) (Karim et al., 2014b), but was lower than that in captive golden snub-nosed monkeys (46.2%, 74/160) (Yu et al., 2017), and captive Asiatic black bears (27.4%, 29/106) (Yang et al., 2015) in other provinces of China. This is most likely due to the worse raising conditions and generally higher density of minks in investigated farms. We found that minks from Hebei province (32.3%, 95% CI 14.8–49.7, OR = 7.05, *P* = 0.01) have a higher prevalence of *E. bieneusi* compared with Jilin (10.5%, 95% CI 2.9–18.0, OR = 1.7), Heilongjiang (6.3%, 95% CI 0.8–11.8, OR = 1), Liaoning (8.9%, 95% CI 2.9–14.9, OR = 1.4), and Shandong provinces (0%). Moreover, the difference in prevalence may be related to other factors, such as animal welfares, climates, and animal husbandry practices.

More than 50 *E. bieneusi* ITS genotypes have been identified in captive animals in China (**Table 4**). However, only five genotypes were identified in present study. These findings suggested that the five genotypes were endemic *E. bieneusi* in minks in Northern China. In the present study, the most prevalent genotype was D which has also been found in captive non-human primates, domestic rabbits, captive foxes, captive raccoon dogs, pet chinchillas, and many other captive wildlife (Karim et al., 2014a; Du et al., 2015; Qi et al., 2015; Yang et al., 2015, 2016, 2017; Zhao et al., 2015; Li et al., 2016b; Xu et al., 2016; Zhang et al., 2016a,b; Yu et al., 2017). In addition, the second most prevalent genotype was EbpC which was also more frequently found in some captive animals (captive foxes and captive non-human primates) (Karim et al., 2014a; Zhao et al., 2015; Yang et al., 2017); however, Peru11 was only found in captive non-human primates in China (Karim et al., 2014a; Yang et al., 2017). These findings also suggested that these genotypes of *E. bieneusi* might transmission among these captive animals. More importantly, three known genotypes of *E. bieneusi* from the present study (D, Peru11, and EbpC) have been also previously identified in humans in China (Wang et al., 2013; Ojuromi et al., 2016). Therefore, our results have indicated that mink might be a potential source of infection for humans. Moreover, seven polymorphic sites were observed among the five genotypes, implying the more genetic diversity of *E. bieneusi* in minks in Northern China.

**Table 4:** Prevalence of *Enterocytozoon bieneusi* infection in caged animals in China.

Regions	Hosts	Genotypes	Prevalence (no. positive/no. tested)	Reference
Shaanxi	Captive non-human primates	D, BEB6, MH, XH, BSH	12.7% (25/192)	Du et al., 2015
Henan, Guangxi, Sichuan, Yunnan, and Guangdong	Non-human primates	Type IV, D, Henan V, Peru8, PigEBITS7, EbpC, Peru11, BEB6, I, CM1 to CM7	11.4% (158/1386)	Karim et al., 2014a
Jilin and Liaoning	Domestic rabbits	D	0.9% (4/426)	Zhang et al., 2016b
Heilongjiang	Rabbits	CHN-RD1, D, Type IV, Peru6, I, CHN-RR1 to CHN-RR3	10.2% (22/215)	Yang et al., 2016
Jilin, Heilongjiang, and Hebei	Foxes	Peru 8, Types IV, CHN-DC1, D, NCF1 to NCF7	12.3% (37/302)	Zhang et al., 2016a
Heilongjiang	Foxes	D	27.7% (53/191)	Yang et al., 2015
Heilongjiang and Jilin	Blue foxes	D, EbpC, CHN-F1	16.4% (18/110)	Zhao et al., 2015
Jilin, Hebei, Liaoning, Shandong, and Heilongjiang	Raccoon dogs	D, CHN-DC1, NCF2, CHN-F1, NCR1, NCR2	22.3% (68/305)	Xu et al., 2016
Heilongjiang and Jilin	Raccoon dogs	D, CHN-R1	4.1% (2/49)	Zhao et al., 2015
Heilongjiang	Raccoon dogs	D, CHN-DC1, CHN-DC1/WildBoar3	10.5% (17/162)	Yang et al., 2015
Beijing, Zhengzhou, Anyang, and Guiyang	Pet chinchillas	BEB6, D	3.6% (5/140)	Qi et al., 2015
Guangxi	Captive snakes	Type IV, Henan V, CRep-1 to CRep-4	4.6% (11/240)	Karim et al., 2014b
Chengdu	Captive wildlife	D, Peru 6, CHB1, BEB6, CHS9, SC02, SC03	15.8% (43/272)	Li et al., 2016b
Sichuan and Guizhou	Captive Asiatic black bears	CHB1, SC02, horse2, ABB1 and ABB2	27.4% (29/106)	Deng et al., 2017
Beijing, Shanghai, Anhui, and Shanxi	Captive golden snub-nosed monkey	D, J, CHG1, CHG14, CM19 to CM 21	46.2% (74/160)	Yu et al., 2017
Beijing	Laboratory macaques	CC4, CM1, CM2, D, Peru8, Peru11, Type IV, WL21, CMB1, CMB2	18.0% (37/205)	Yang et al., 2017
Jilin, Hebei, Liaoning, Shandong, and Heilongjiang	Minks	D, Peru11, EbpC, and two novel genotypes NCM-1 and NCM-2		This study


Minks is one of the most important economic animals. They can provide thick fur for humans. Therefore, more and more minks were raised as an important source of income in many countries. Previous studies have indicated that many pathogens can infect minks (Chalmers et al., 2015; Gholipour et al., 2017; Xie et al., 2018). In the present study, we found that *E. bieneusi* can also infect minks which expend the hosts range of *E. bieneusi*. The results of the present survey have implications for the controlling *E. bieneusi* infection in minks, other animals and humans in China and elsewhere in the world.

## Conclusion

This is the first record of *E. bieneusi* in minks, and the prevalence is associated with the region of investigated minks. These findings also suggest that D, Peru11, EbpC, NCM-1, and NCM-2 are endemic in minks in Northern China. The occurrence of zoonotic *E. bieneusi* genotypes in the feces of the minks suggests potential environmental contamination with *E. bieneusi* oocysts and may raise a public health concern. Moreover, effective measures should be implemented to avoid water-born microsporidiosis outbreaks.

## Data Availability Statement

Representative nucleotide sequences were submitted to GenBank under accession numbers: MF440664–MF440668.

## Author Contributions

G-HL and GH conceived and designed the study, and critically revised the manuscript. X-XZ, R-LJ, and J-GM performed the experiments. X-XZ and R-LJ analyzed the data. X-XZ drafted the manuscript. CX and QZ helped in study design, study implementation, and manuscript preparation. All authors read and approved the final manuscript.

## Conflict of Interest Statement

The authors declare that the research was conducted in the absence of any commercial or financial relationships that could be construed as a potential conflict of interest.
